# Clinicopathological Features, Management, and Outcomes of Zebra Spitting Cobra (*Naja nigricincta nigricincta*) and Puff Adder (*Bitis arietans*) Snakebites in Namibia

**DOI:** 10.1155/bmri/6571833

**Published:** 2026-07-24

**Authors:** Katrina Ndongi, Emmanuel Nepolo, Christian J. Hunter

**Affiliations:** ^1^ Department of Human Biological and Translational Medical Sciences, University of Namibia, Windhoek, Namibia, unam.edu.na; ^2^ Clinical Care, Education and Research, Centre of Global Health Practice and Impact, Georgetown University, Washington, DC, USA, georgetown.edu

**Keywords:** antivenom, *Bitis arietans*, cytotoxicity, *Naja nigricincta nigricincta*, puff adder, rhabdomyolysis, snakebite envenomation, zebra spitting cobra

## Abstract

Snakebite envenomation remains an important public health concern in Namibia, particularly following bites from the zebra spitting cobra (*Naja nigricincta nigricincta*) and the puff adder (*Bitis arietans*). However, the clinical presentations and management outcomes of these snakebites remain poorly characterized. This prospective, observational, descriptive study investigated the clinicopathological features, wound progression, management, and functional outcomes of snakebite patients treated at two major referral hospitals in Namibia. A total of 20 hospitalized patients were included. The majority of patients were male (65%) and children aged 0–15 years (65%). Zebra spitting cobra bites accounted for 60% of cases. Eighty percent of patients presented with predominantly cytotoxic envenomation. Neurological symptoms were documented in a small number of patients (33% of zebra spitting cobra cases and 13% of puff adder cases), but were mild and nonspecific. Antivenom was administered to only 15% of patients. Zebra spitting cobra envenomation was associated with laboratory abnormalities consistent with systemic involvement such as anemia, hemolysis, rhabdomyolysis, hepatic involvement, and renal impairment, whereas puff adder bites demonstrated minimal systemic involvement. Surgical intervention was frequently required, including debridement, fasciotomy, and amputations (15%); however, no fatalities were recorded. These findings highlight the substantial morbidity associated with snakebite envenomation in Namibia and emphasize the importance of early referral, appropriate wound management, and improved access to effective antivenom. However, these findings should be interpreted within the limitations of the small sample size, convenience sampling, and incomplete laboratory investigations in some patients. Nevertheless, this study provides important baseline clinical data to support future snakebite management strategies and region‐specific treatment guidelines in Namibia.

**Key Contribution**

This study represents one of the first detailed clinical and laboratory descriptions of zebra spitting cobra (*Naja nigricincta nigricincta*) and puff adder (*Bitis arietans*) envenomation in Namibia, highlighting patterns of local tissue pathology, systemic laboratory abnormalities, surgical management, and functional outcomes in hospitalized patients.

## 1. Introduction

Snakebite envenomation is a neglected tropical disease that predominantly affects rural and marginalized populations with limited access to healthcare and antivenom across sub‐Saharan Africa [1–4]. Globally, snakebites account for 1.8–2.7 million cases of envenomation and up to 90,000 deaths annually, with an estimated 400,000 people left with permanent disabilities, including amputations [2, 4, 5]. Asia has the highest burden of snakebite envenomation, with an estimated 1.2–2.0 million cases annually, followed by Africa, which experiences approximately 435,000–580,000 cases and 20,000–32,000 deaths each year [5]. The World Health Organization (WHO) recognizes snakebite envenomation as a high‐priority public health concern, targeting a 50% reduction in deaths and disabilities by 2030 [2, 6].

In Namibia, an estimated 572–811 snakebite cases occur annually, resulting in 25–38 deaths and 23–65 amputations [7, 8]. Although snakebite envenomation occurs throughout Namibia, the burden is greatest in rural areas, where limited access to specialized care often necessitates referral of patients with severe envenomation to tertiary hospitals in Windhoek for antivenom therapy, surgical management, and intensive supportive care [9]. A snake‐removal survey conducted in Windhoek identified the puff adder (*B. arietans*, 163 removals) and the zebra spitting cobra (*N. nigricincta nigricincta*, 135 removals) as the two most commonly encountered species in the region, and both are recognized as medically important due to their potential to cause severe local and systemic envenomation [9].

Despite the burden of snakebite envenomation in Namibia, there is limited published clinical evidence describing the pathology, management, and outcomes associated with bites from medically important snake species [10]. Both the zebra spitting cobra and puff adder produce predominantly cytotoxic venom associated with severe local tissue injury and necrosis [11, 12]. Zebra spitting cobra envenomation has also been associated with neurological symptoms and laboratory abnormalities suggestive of systemic involvement, whereas puff adder envenomation more commonly presents with predominantly local tissue injury, although systemic manifestations have been reported in severe cases [11, 12].

Current treatment protocols rely largely on WHO and Ministry of Health and Social Services (MoHSS) guidelines and emphasize supportive care, timely surgical intervention when clinically necessary, and antivenom administration when available [13, 14]. However, access to antivenom remains limited, and evidence regarding its effectiveness against locally important species is scarce [14]. To help address this gap, this study documents wound progression, systemic laboratory abnormalities, treatment interventions, and patient outcomes, providing baseline evidence to support clinical management, guide future research, and strengthen snakebite care in Namibia.

## 2. Methods

### 2.1. Study Design and Setting

This prospective, observational, descriptive study was conducted at Katutura Intermediate Hospital (KISH) and Windhoek Central Hospital (WCH) in Windhoek, Namibia. Both hospitals serve as the secondary and tertiary referral centers for patients from across Namibia, respectively. Patients managed exclusively at primary healthcare facilities or on an outpatient basis were excluded. Data were collected over a 15‐month period from December 2020 to March 2022.

### 2.2. Study Population

The study included only snakebite cases attributed to the zebra spitting cobra (*N. nigricincta nigricincta*) and the puff adder (*B. arietans*); cases involving unidentified or other species were excluded. Species identification was established using a multimodal approach based on patient or witness reports. In some cases, patients or witnesses provided photographs of the snake taken at the scene, whereas in others, the snake responsible for the bite was killed and brought to the hospital for direct examination. Species identification was supported using standardized snake identification posters [15].

A total of 20 patients were enrolled using convenience sampling. This sampling approach was chosen because snakebite cases, especially those with confirmed species identification, were encountered unpredictably throughout the study period.

### 2.3. Data Collection

Data were collected by the principal investigator using three structured, study‐specific instruments. In the absence of a universally accepted standardized data collection form for snakebite envenomation, these tools were developed based on a comprehensive review of relevant clinical literature [1, 3, 11, 13, 14, 16–19]. The patient interview tool, administered within 48 h of admission, captured demographic information, clinical history, and physical examination findings. A clinical monitoring tool was used to document daily assessments of wound progression from admission until discharge. A laboratory data extraction tool was used to retrieve relevant findings from patient records.

#### 2.3.1. Laboratory Assessments

Patient management adhered to standard Namibian snakebite treatment protocols, with laboratory investigations requested by the treating clinical team according to clinical indication [13, 14]. Hematological assessments included full blood count, blood smears to evaluate laboratory abnormalities suggestive of anemia and hemolysis. Biochemical investigations included serum urea and creatinine to assess renal impairment; alanine transaminase (ALT) and aspartate transaminase (AST) to assess hepatic involvement; and creatine kinase (CK), lactate dehydrogenase (LDH), CK‐MB, and myoglobin to evaluate muscle injury and rhabdomyolysis [13, 14].

#### 2.3.2. Definition of Clinical Conditions

A “dry bite” was defined as a bite by a venomous snake characterized by puncture wounds (fang marks) but a complete absence of local or systemic envenomation symptoms and laboratory abnormalities during a minimum observation period of 24 h [20]. Envenomation was defined by the presence of local clinical manifestations, such as pain, swelling, blistering, or tissue necrosis, and/or systemic manifestations. These systemic features included neurological symptoms like ptosis (drooping of the upper eyelid), diplopia (blurred or double vision), dysphagia (inability to swallow saliva), limb weakness, or respiratory muscle paralysis (respiratory muscle weakness leading to respiratory paralysis), as well as laboratory abnormalities consistent with envenomation [11, 12].

Standardized clinical and laboratory criteria were employed to define the systemic manifestations of envenomation. Anemia was defined according to WHO reference ranges as a hemoglobin concentration < 12.0 g/dL for females and < 13.0 g/dL for males [21]. Hemolysis was considered present when decreases in hemoglobin and hematocrit were accompanied by laboratory abnormalities suggestive of red cell destruction, including elevated LDH and bilirubin levels [3, 19, 22].

Rhabdomyolysis was defined as a serum CK concentration exceeding five times the upper limit of normal (> 1000 U/L). Hepatic involvement was defined as ALT and/or AST concentrations greater than two times the upper limit of normal (> 80 U/L) [22]. Acute kidney injury (AKI) was defined and staged according to the Kidney Disease: Improving Global Outcomes (KDIGO) clinical practice guidelines [23], as adapted by local protocols [3, 19, 24]. AKI was defined by an increase in serum creatinine of ≥ 26.5 *μ*mol/L (0.3 mg/dL) within 48 h, an increase of ≥ 50% from baseline within 7 days, a documented decrease in glomerular filtration rate (GFR), or a urine output of < 0.5 mL/kg/h for more than 6 consecutive hours.

#### 2.3.3. Clinical Outcomes Assessed

Clinical outcomes assessed during hospitalization included local pathological effects, systemic manifestations of envenomation, and functional outcomes at discharge. The specific variables evaluated within each domain are summarized in Table 1.

**Table 1:** Outcome measures assessed during hospitalization.

Outcome domain	Variables assessed
Local pathology	Swelling, blistering, necrosis, and requirement for surgical intervention.
Systemic findings	Anemia, hemolysis, rhabdomyolysis, renal impairment, hepatic involvement, and neurological symptoms.
Functional outcomes at discharge	Complete recovery, persistent muscle weakness, and amputation.

### 2.4. Statistical Analysis

Data were analyzed using GraphPad Prism Version 8.0.2 (GraphPad Software, San Diego, California, United States). Descriptive statistics were used to summarize patient demographics, clinical characteristics, laboratory findings, treatment interventions, and outcomes. Continuous variables were assessed for normality using the Shapiro–Wilk test; these were presented as means ± standard deviations (SD) for normally distributed data or medians and interquartile ranges (IQR) for nonnormally distributed data. Categorical variables were presented as frequencies and percentages. Given the small sample size and observational nature of the study, analyses were primarily descriptive, and formal comparative statistical testing was not performed.

### 2.5. Ethical Considerations

The study was approved by the University of Namibia Health Research Ethics Committee (H‐G/384/2020) and the MoHSS Ethics Committee (17/3/3/KN). All patients or guardians provided written informed consent. Privacy and confidentiality were maintained by coding identifiers and anonymizing images and data.

## 3. Results

### 3.1. Patient Demographics and Epidemiology

Between December 2020 and March 2022, 20 snakebite patients were admitted to KISH and WCH following bites by either a zebra spitting cobra (*N. nigricincta nigricincta*) or a puff adder (*B. arietans*). Of the 20 enrolled patients, two (10%) were classified as dry bites.

The median age of patients was 10 years (IQR 4–27). Children aged 0–15 years accounted for 65% of cases. Most patients (85%) were referred from rural regions. Snakebites also occurred predominantly during the summer season, accounting for 85% of cases. Demographic and epidemiological characteristics stratified by snake species are presented in Table 2.

**Table 2:** Demographic and epidemiological characteristics of patients with zebra spitting cobra (*Naja nigricincta nigricincta*) and puff adder (*Bitis arietans)* envenomation.

Population characteristics	Zebra spitting cobra (*n*, %)	Puff adder (*n*, %)
Age		
0–15 years	7 (58.3)	6 (75.0)
16–30 years	4 (33.3)	—
31–45 years	1 (8.3)	2 (25.0)
Gender		
Male	9 (75.0)	4 (50.0)
Female	3 (25.0)	4 (50.0)
Bite site (body)		
Head	5 (41.6)	—
Lower extremities	2 (16.6)	4 (50.0)
Upper extremities	4 (33.3)	3 (37.5)
Abdomen	—	1 (12.5)
Genitalia	1 (8.3)	—
Season of snakebite		
Summer	11 (91.6)	6 (75.0)
Autumn	—	1 (12.5)
Winter	—	1 (12.5)
Spring	1 (8.3)	—
Activity at time of bite		
Sleeping	9 (75.0)	1 (12.5)
Walking	—	3 (37.5)
Sitting	1 (8.3)	1 (12.5)
Playing	1 (8.3)	3 (37.5)
Standing	1 (8.3)	—
Location of the bite		
Indoor	10 (83.3)	1 (12.5)
Outdoor	2 (16.7)	7 (87.5)
Severity of swelling		
Severe	10 (83.3)	2 (25.0)
Moderate	2 (16.6)	2 (25.0)
Mild	—	2 (25.0)
None	—	2 (25.0)
Antivenom administration		
Yes	1 (8.3)	2 (25.0)
No	11 (91.7)	6 (75.0)

*Note:* Snake species: zebra spitting cobra (*n* = 12) and puff adder (*n* = 8). Antivenom was administered in three patients.

### 3.2. Bite‐to‐Care Interval

Snakebites occurred predominantly at night, with 14 of 20 cases (70%) occurring between 18:00 and 05:59. The highest frequencies were observed at 23:00 and 02:00, with each time point accounting for three patients (15%). The median time of the bite was 23:00 (IQR 22:00–02:00) for zebra spitting cobra cases and 14:00 (IQR 10:00–17:00) for puff adder bites. Following snakebite, the median interval before presentation at healthcare facilities was 1.5 h (IQR 1.0–3.5 h). Additionally, most patients received initial care at primary facilities before being transferred to KISH and WCH.

### 3.3. Local Envenomation and Wound Progression

Rapid local pain and swelling were observed in 18 of the 20 patients (90%), comprising 12 severe, four moderate, and two mild presentations. All affected patients presented to healthcare facilities—ranging from primary clinics to tertiary referral centers—within 6 h of the bite. During the first 24–48 h, laboratory abnormalities suggestive of rhabdomyolysis, hemolysis, hepatic involvement, and, in one case, AKI became apparent in patients with zebra spitting cobra envenomation. Between Days 3 and 6, tissue necrosis had become clearly demarcated, allowing definitive surgical assessment. Fasciotomy and debridement were performed during the first week of admission, with repeated debridement and skin grafting required in selected patients. Length of hospital stay ranged from 1 to 43 days (mean 18 ± 14 days).

Tissue necrosis occurred in 11 of the 12 zebra spitting cobra cases (92%) and four of the eight puff adder cases (50%). Blistering was observed in 33% (*n* = 4) of zebra spitting cobra cases and 25% (*n* = 2) of puff adder cases. Clinically suspected compartment syndrome was more frequent in zebra spitting cobra bites, occurring in 58% (*n* = 7) of cases, compared with 25% (*n* = 2) of puff adder cases. These severe local effects frequently resulted in significant tissue loss, with most patients ultimately requiring surgical intervention (detailed in the Management and Surgical Interventions section below).

Traditional remedies were used by four patients prior to hospital presentation, including the application of petrol or methylated spirits and incision of the bite site. Although zebra spitting cobras are capable of defensive venom spraying, no cases of ocular envenomation were identified during the study period.

**Figure 1:**
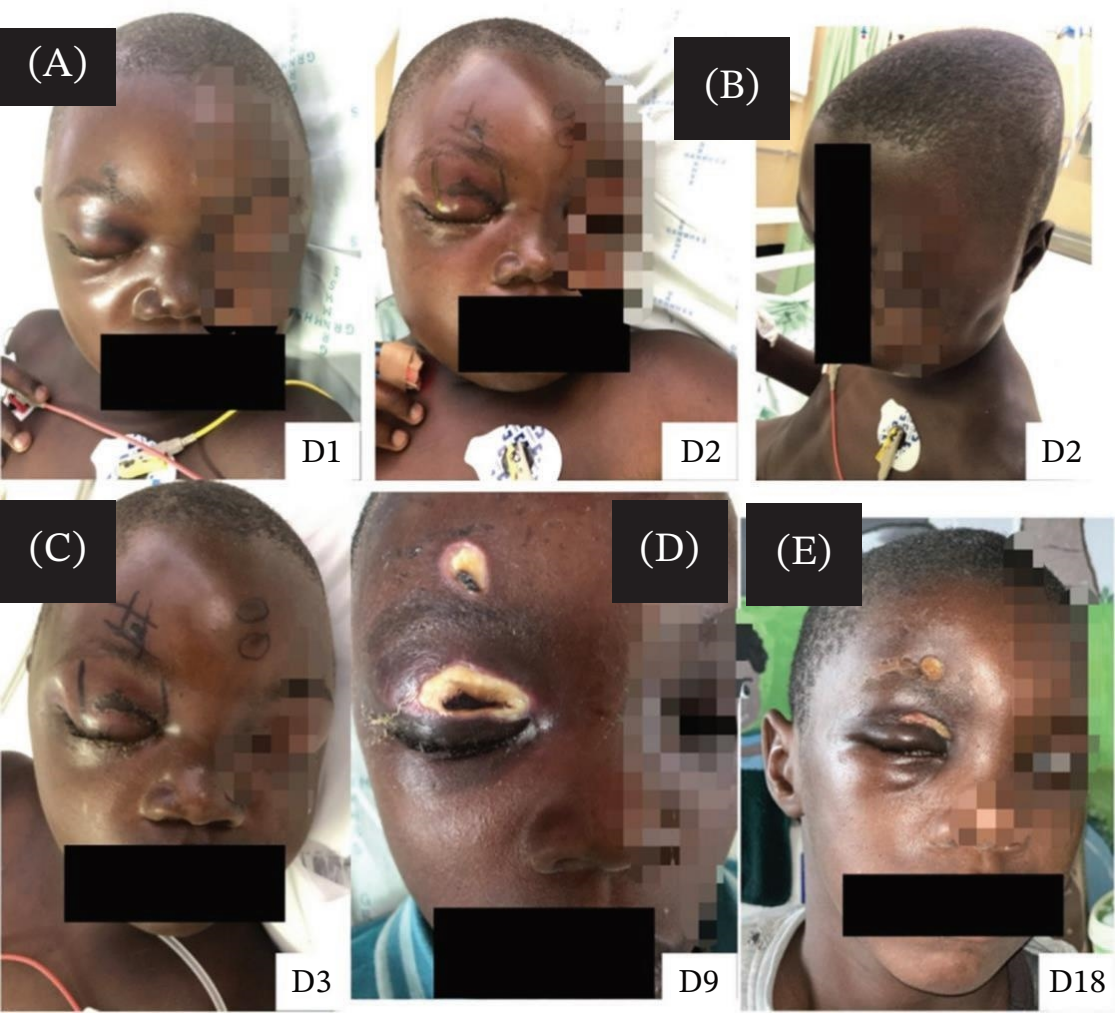
Progression of facial necrosis following zebra spitting cobra envenomation in Case 1. Antivenom was not administered. (A) Day 1 (D1): severe facial edema; (B) Day 2: necrotic tissue above the right eye; (C) Day 3: progressive facial swelling with continued tissue necrosis; (D) Day 9: surgical debridement; and (E) Day 18: recovery following discharge.

**Figure 2:**
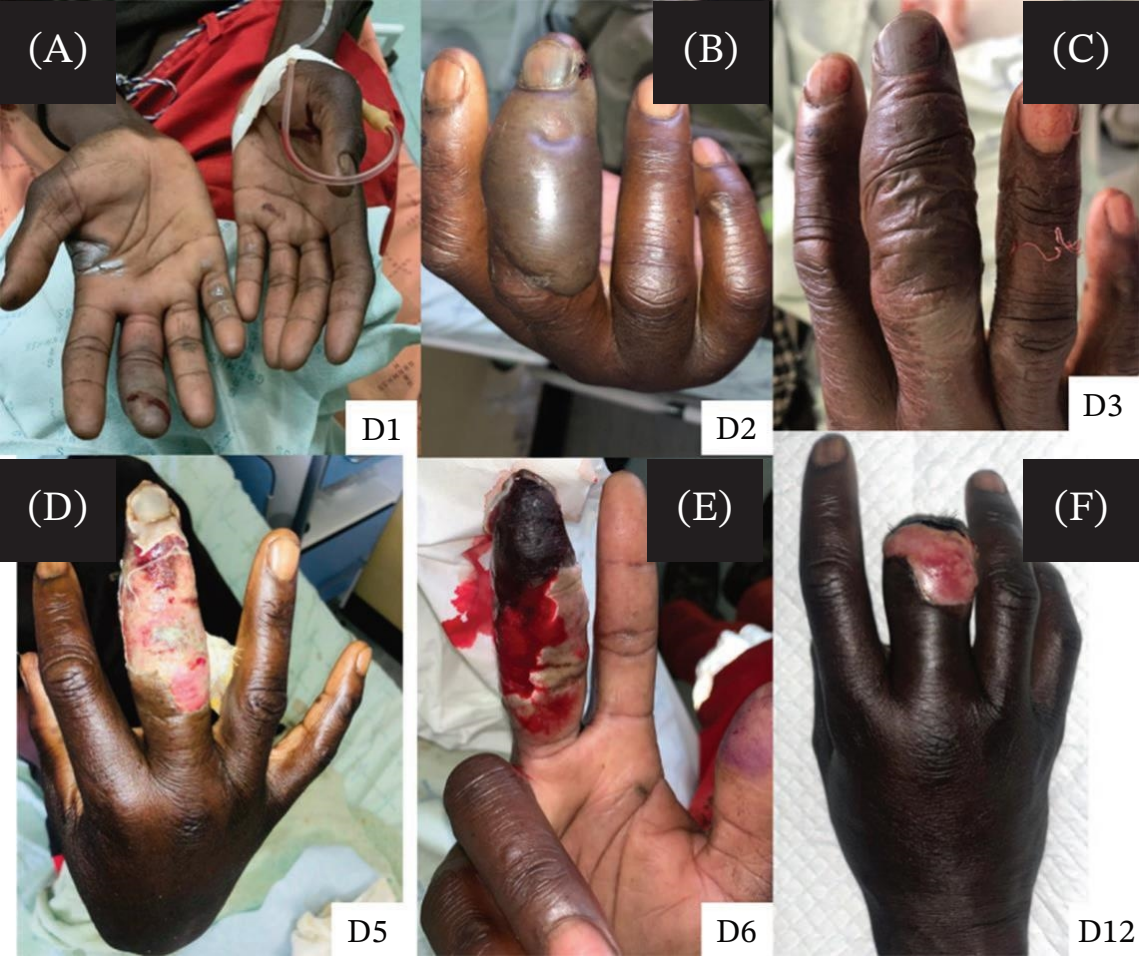
Progression of right index finger necrosis following puff adder envenomation in Case 3. Antivenom was administered. (A) Day 1: mild edema at the bite site; (B) Day 2: severe swelling with fluid accumulation; (C) Day 3: fluid drainage; (D) Day 5: early tissue necrosis; (E) Day 6: progressive necrosis involving the distal phalanx; and (F) Day 12: amputation of the affected digit.

**Figure 3:**
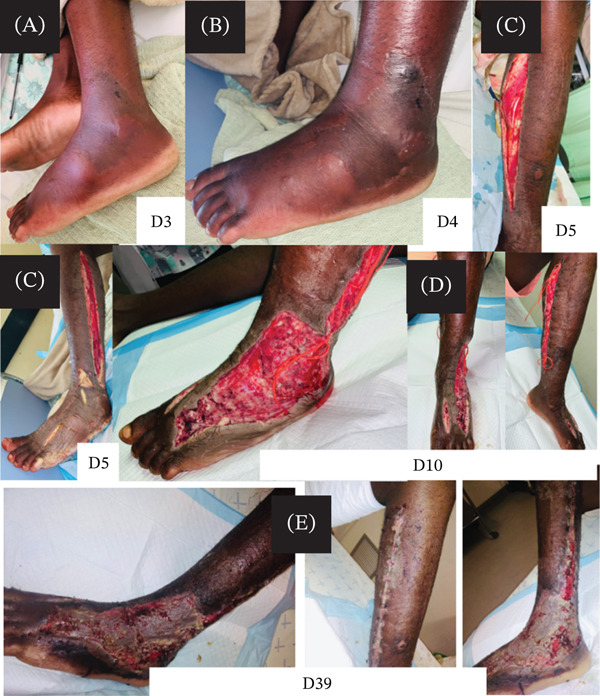
Progression of severe left foot necrosis following zebra spitting cobra envenomation in Case 5. Antivenom was not administered. (A) Day 3: marked edema and tissue necrosis surrounding the fang marks; (B) Day 4: blister formation; (C) Day 5: fasciotomy and surgical debridement; (D) Day 10: reduced swelling; and (E) Day 39: post‐skin graft healing.

**Figure 4:**
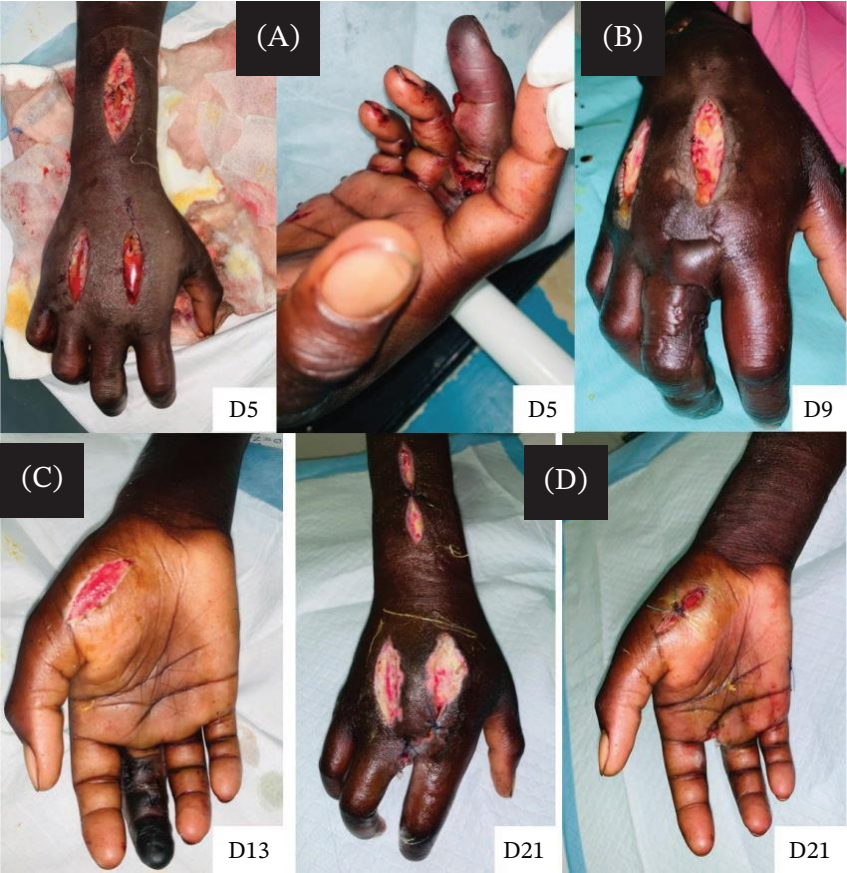
Progression of right middle finger necrosis following zebra spitting cobra envenomation in Case 20. Antivenom was not administered. (A) Day 5: postfasciotomy and surgical debridement; (B) Day 9: blister formation; (C) Day 13: extensive necrosis involving the entire finger; and (D) Day 21: postamputation healing.

Representative examples of wound progression and tissue necrosis are shown in Figures 1, 2, 3 and 4. These cases illustrate the spectrum of local tissue injury observed following envenomation by zebra spitting cobra and puff adder, including progressive necrosis, blister formation, fasciotomy, debridement, skin grafting, and amputation.

### 3.4. Systemic Envenomation

Systemic complications, beyond mild neurological symptoms, were observed exclusively in the zebra spitting cobra cohort. Conversely, puff adder cases were characterized by localized pathology and lacked laboratory evidence of systemic envenomation.

#### 3.4.1. Clinical Presentation: Vital Signs, Hemodynamic Profile, and Neurological Symptoms

At presentation, patients were hemodynamically stable. Systolic blood pressure ranged from 102 to 150 mmHg, with no evidence of clinical shock. However, autonomic stress responses were prevalent: 35% of patients (7/20) presented with tachycardia (87–145 bpm) and 15% (3/20) with tachypnea (16–34 bpm). Peripheral oxygen saturation (SpO_2_) ranged from 92% to 100% across all cases.

Neurological symptoms were observed in 25% (*n* = 5) of the study cohort, comprising one (13%) puff adder and four (33%) zebra spitting cobra envenomation cases (Table 3). The reported neurological symptoms included limb weakness (*n* = 3), respiratory muscle weakness (*n* = 3), diplopia (*n* = 2), and dysphagia (*n* = 1).

**Table 3:** Neurological symptoms among patients with snakebite envenomation.

Neurological symptoms	Case 4	Case 6	Case 10	Case 17	Case 19
Limb weakness	●	●	●		
Respiratory muscle weakness	●	●	●		●
Dysphagia				●	
Diplopia	●				●
Snake species	PA	ZSC	ZSC	ZSC	ZSC

*Note:* The symbol “●” indicates present symptom. Blank cells indicate that the specific neurological symptom was absent. None of these patients received antivenom.

Abbreviations: PA, puff adder; ZSC, zebra spitting cobra.

#### 3.4.2. Biochemical and Hematological Laboratory Findings

Laboratory abnormalities in the zebra spitting cobra cohort became clinically apparent within 24–48 h of admission (Table 4). Rhabdomyolysis was the most frequently observed systemic abnormality, affecting seven of the 12 patients (58%), followed by anemia and laboratory findings suggestive of hemolysis, each occurring in six patients (50%). Hepatic involvement was documented in four patients (33%), whereas AKI was observed in only one patient (8%) and resolved following intravenous fluid therapy.

**Table 4:** Biochemical and hematological abnormalities observed in 12 zebra spitting cobra envenomation cases.

Condition	Patients, *n* (%)	Key laboratory findings
Anemia	6 (50)	Acute declines in hemoglobin and hematocrit during hospitalization. Reticulocyte counts, where available, remained within the reference range.
Laboratory findings suggestive of hemolysis	6 (50)	Declining hemoglobin accompanied by elevated LDH and bilirubin. Confirmatory investigations, including direct antiglobulin (Coombs′) testing, were not performed.
Rhabdomyolysis	7 (58)	Elevated creatine kinase (CK) and LDH concentrations associated with extensive local tissue necrosis.
Hepatic involvement	4 (33)	ALT and/or AST > 2x the upper limit of normal (AST: 98–451 U/L; ALT: 88–90 U/L). Albumin and ALP remained within reference ranges, with no evidence of hepatic failure.
Acute kidney injury (AKI)	1 (8)	Transient elevation in serum creatinine and urea that resolved with intravenous fluid therapy.

Hematological and biochemical markers were monitored to assess systemic envenomation. In Case 1, hemoglobin levels decreased, accompanied by elevated LDH and bilirubin; peripheral blood smear examination revealed normocytic, normochromic anemia, spherocytes, and neutrophils with toxic granulation. Case 3 presented with transient neutrophilic leukocytosis, and renal parameters remained within reference ranges throughout hospitalization. Evidence of muscle injury was noted in Case 5, where CK increased from 573 U/L on admission to 1712 U/L by Day 3, alongside transient prolongation of PT and INR. This was followed by a decline in hemoglobin and hematocrit, whereas renal function remained stable. In Case 20, hemoglobin decreased from within the reference range on Day 4 to 10.7 g/dL on Day 7, concurrent with leukocytosis, neutrophilia, thrombocytosis, and elevated LDH. Peripheral blood smear examination and reticulocyte counts were not performed for this patient.

The distribution of systemic manifestations among zebra spitting cobra cases is summarized in Table 5. Puff adder cases were excluded because no significant laboratory abnormalities were identified.

**Table 5:** Systemic clinical features and antivenom administration among zebra spitting cobra snakebite cases.

Clinical feature	Case 1	Case 5	Case 8	Case 9	Case 13	Case 15	Case 17	Case 19	Case 20
Anemia	●	●				●	●	●	●
Hemolysis	●			●	●	●	●		●
Rhabdomyolysis	●		●	●	●	●	●	●	
Renal impairment						●			
Hepatic involvement	●		●		●			●	
Antivenom administered	No	No	No	Yes	No	No	No	No	No

*Note:* The symbol “●” indicates presence of the clinical feature. Blank cells indicate that the specific clinical feature was absent. Systemic findings were observed only in cases attributed to zebra spitting cobra, whereas puff adder cases demonstrated no significant laboratory abnormalities. Antivenom was administered in a limited number of cases overall.

### 3.5. Management and Surgical Interventions

Intravenous fluids, pain medication, antibiotics, and limb elevation were used as supportive care. Antibiotic (clindamycin, augmentin, ceftriaxone, cloxacillin, and cefuroxime) therapy was administered only to patients who developed tissue necrosis or clinical evidence of wound infection. In total, three patients (15%) were administered polyvalent antivenom from the South African Institute for Medical Research (SAIMR). Two patients achieved complete recovery, one following a zebra spitting cobra bite and the other after a puff adder bite, whereas the third patient with a puff adder bite underwent surgical debridement and amputation.

Local tissue injury frequently necessitated surgical management. Overall, 12 patients (60%) required one or more surgical procedures. Of the full cohort (*n* = 20), this resulted in 12 debridements (60%), seven fasciotomies (35%), four skin grafts (20%), and three amputations (15%) (Table 6). To relieve elevated compartmental pressure, approximately 42% (*n* = 5) of patients bitten by the zebra spitting cobra underwent fasciotomy, whereas 25% (*n* = 2) of those bitten by the puff adder required the procedure. Debridement of necrotic tissue was performed on nine of the patients bitten by zebra spitting cobra and three of the patients bitten by puff adder. Of the three patients who received antivenom, only two required surgical intervention.

**Table 6:** Surgical interventions in the patients.

Surgical intervention	Case 1	Case 3	Case 5	Case 6	Case 7	Case 8	Case 9	Case 10	Case 12	Case 15	Case 19	Case 20
Fasciotomy		✓		✓		✓		✓		✓	✓	✓
Debridement	✓	✓	✓	✓	✓	✓	✓	✓	✓	✓	✓	✓
Skin graft			✓	✓				✓			✓	
Amputation		✓		✓								✓
Snake species	ZSC	PA	ZSC	ZSC	PA	ZSC	ZSC	ZSC	PA	PA	ZSC	ZSC
Antivenom administered	No	Yes	No	No	No	No	Yes	No	No	No	No	No

*Note:* The symbol “✓” indicates procedure performed. Blank cells indicate that the procedure was not performed. Antivenom administration status indicates whether antivenom therapy was administered during patient management.

Abbreviations: PA, puff adder; ZSC, zebra spitting cobra.

### 3.6. Patient Outcomes

**Figure 5:**
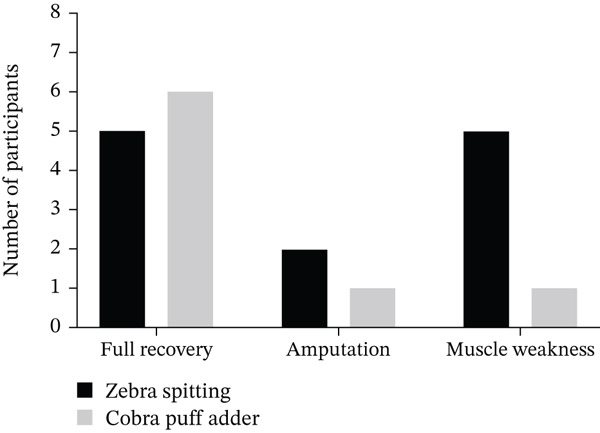
Functional outcomes among patients with zebra spitting cobra (*n* = 12) and puff adder (*n* = 8) envenomation. Bar chart showing proportion of patients with complete recovery, persistent muscle weakness, and limb amputation. Antivenom was administered in three cases only.

Patients with moderate envenomation were typically discharged within approximately 18 days, whereas severe cases requiring extensive wound management remained hospitalized for up to 43 days. Complete recovery was achieved in 55% (11/20) of patients, whereas 30% (6/20) were discharged with persistent muscle weakness and 15% (3/20) underwent amputation. Amputations occurred in two zebra spitting cobra cases and one puff adder case (Figure 5).

## 4. Discussion

### 4.1. Epidemiological Profile

The zebra spitting cobra (*N. nigricincta nigricincta*) was the most frequently implicated species in this study, accounting for 60% of all snakebite cases. Moreover, snakebite occurred most frequently among children and males during the summer months, a demographic pattern consistent with findings from other sub‐Saharan African studies [3, 11, 16, 17, 19]. The higher incidence among males likely stems from their greater involvement in outdoor activities such as farming and herding. This increases their risk of accidental snake encounters. Similarly, children are particularly vulnerable because they spend more time playing outdoors and may be less aware of potential environmental hazards.

The circumstances and anatomical sites of snakebite differed between zebra spitting cobra and puff adder (*B. arietans*) envenomation cases, reflecting the behavior of these species. Zebra spitting cobra bites occurred mainly indoors and while victims were sleeping, which likely explains the high frequency of bites involving the face, head, and upper extremities. The predominance of indoor bites during sleep suggests that housing conditions may contribute to envenomation in rural communities. In contrast, puff adder bites were primarily associated with outdoor activities, such as walking or herding, which aligns with their ambush‐predatory, ground‐dwelling behavior [25, 26]. This differentiation is clinically relevant as it suggests that preventive strategies should be tailored, emphasizing indoor “snake‐proofing” and bed‐net use for zebra spitting cobras, while promoting protective footwear and vigilance during outdoor labor to prevent puff adder encounters.

The use of traditional remedies prior to hospital presentation, observed in several patients, may have contributed to delays in seeking definitive medical care, a practice previously reported in other snakebite‐endemic regions [3, 11, 19]. Community education regarding appropriate first‐aid measures and the need for prompt presentation to healthcare facilities following snakebite should be strengthened and sustained.

### 4.2. Local Tissue Pathology in Snakebite Patients

The clinical differences observed between zebra spitting cobra and puff adder envenomations likely reflect variations in venom composition, fang morphology, and delivery mechanisms. The zebra spitting cobra′s short, fixed fangs tend to deposit venom superficially, producing marked local cytotoxic effects and necrosis [11]. Although this species can induce neurotoxicity, its venom is predominantly characterized by potent cytotoxins that contribute to local tissue damage. In contrast, the puff adder′s long, hinged solenoglyphous fangs facilitate deep tissue penetration and venom delivery into muscle compartments, resulting in extensive swelling, compartment involvement, and severe local injury [11, 12]. Accordingly, puff adder envenomation in this cohort was characterized predominantly by severe local tissue injury, with no clinical or laboratory evidence of systemic envenomation. These disparities in venom composition and delivery likely contribute to the distinct clinicopathological manifestations observed in this study.

Consistent with previous research, 80% of patients presented with local manifestations such as pain, swelling, blistering, and tissue damage [2, 3, 11, 12, 19]. The extensive tissue necrosis observed in this study is consistent with previous studies demonstrating that the venoms of zebra spitting cobra and puff adder contain cytotoxic components capable of inducing severe local tissue injury [8, 11]. Overall, 60% of patients required one or more surgical interventions, such as debridement, highlighting the substantial burden of local tissue destruction inherent in cytotoxic envenomation. These findings underscore that local tissue injury, rather than systemic complications, was the principal driver of morbidity in this cohort. This proportion appears higher than that reported in other southern African studies, likely reflecting the referral nature of our study hospitals, which preferentially manage more severe cases of envenomation [11]. Furthermore, fasciotomies were performed based on clinical judgment rather than objective compartment pressure measurements. This management approach deviates from international guidelines, which recommend that objective compartment pressure measurements be used to guide fasciotomy [3, 19, 27].

Although most patients reached a healthcare facility within a relatively short time (median 1.5 h), extensive tissue necrosis and severe local pathology remained common, particularly following zebra spitting cobra envenomation. Despite early presentation, local tissue injury resulting from snakebite envenomation continued to progress over several days, highlighting the importance of close clinical monitoring and timely surgical intervention in patients with severe cytotoxic envenomation.

### 4.3. Systemic Effects From Snake Envenomation

Systemic laboratory abnormalities were observed exclusively following zebra spitting cobra envenomation. Rhabdomyolysis, identified by marked elevations in CK and LDH, was the most frequently identified abnormality and was strongly associated with extensive local tissue destruction, likely reflecting secondary skeletal muscle injury resulting from severe cytotoxic venom effects and tissue necrosis. Similar findings have been described in patients with severe snakebite envenomation and are recognized complications of extensive muscle injury [24].

Anemia and laboratory abnormalities suggestive of hemolysis were also observed in several patients. Although direct antiglobulin testing and other specialized investigations were unavailable, declining hemoglobin concentrations occurring concurrently with elevated LDH and bilirubin levels support the presence of red cell injury. Notably, these findings are consistent with erythrocyte injury during severe envenomation and have been reported previously in cases involving other cytotoxic elapid species [3, 5, 19].

Neurological symptoms observed in this study were generally mild and identified primarily during routine clinical evaluation; these included diplopia, dysphagia, limb weakness, and respiratory muscle weakness. Notably, no patients developed respiratory muscle paralysis, suggesting that severe neurotoxicity is uncommon in this cohort, despite the presence of transient neurological symptoms. These findings are consistent with previous studies [14, 28, 29]. In contrast, the neurological symptoms observed in the single puff adder case in this study likely represent secondary systemic responses such as pain and local inflammation rather than direct venom‐mediated neurological disturbance.

Renal and hepatic involvement appeared to be secondary physiological consequences rather than direct venom‐mediated toxicity. The predominance of AST over ALT elevations suggests that the observed transaminase abnormalities originated primarily from skeletal muscle injury and hemolysis rather than direct hepatotoxicity. Furthermore, the transient renal impairment observed in one patient responded rapidly to supportive care, although the absence of routine urine output monitoring may have underestimated the frequency of renal involvement.

Most laboratory abnormalities became apparent within the first 24–48 h of hospitalization, highlighting the importance of serial laboratory monitoring during the early phase of admission to detect evolving systemic complications and guide timely supportive management. Overall, these systemic abnormalities were more likely secondary consequences of extensive tissue injury, hypovolemia, and systemic inflammation than direct venom‐mediated organ toxicity. Further studies using standardized diagnostic investigations may better define the mechanisms underlying these systemic complications.

### 4.4. Antivenom Use and Clinical Implications

Only a small proportion (15%) of patients received antivenom, reflecting the limited availability, high cost, and uncertain effectiveness of polyvalent products [14, 24]. For this reason, no conclusions regarding its clinical effectiveness can be drawn. Furthermore, no species‐specific antivenom is currently available for zebra spitting cobra envenomation, further limiting the ability to evaluate the benefits of antivenom therapy in this cohort. Consequently, management relied predominantly on supportive care and surgical intervention. Despite the severity of local tissue injury and the occurrence of systemic complications, including rhabdomyolysis, anemia, hemolysis, hepatic involvement, and AKI, no deaths were recorded. This favorable outcome may reflect the multidisciplinary management provided at tertiary referral centers, including intensive supportive care, close clinical monitoring, and timely surgical intervention where indicated.

However, survival was frequently accompanied by substantial morbidity. Overall, 45% of patients were discharged with either persistent muscle weakness or limb amputation, highlighting the substantial long‐term morbidity associated with snakebite envenomation. In addition, hospital stays of up to 43 days illustrate the considerable healthcare burden associated with severe cytotoxic envenomation. These patients frequently required prolonged wound care, repeated surgical procedures, and rehabilitation, placing substantial demands on healthcare resources. Collectively, these findings demonstrate that the burden of snakebite extends beyond mortality and are consistent with reports from other sub‐Saharan African settings, where delayed presentation, severe local tissue injury, and limited access to effective antivenom contribute substantially to long‐term disability [1–3, 19, 24, 30, 31].

### 4.5. Strengths and Limitations

A major strength of this study was the detailed clinical characterization of snakebite envenomation, facilitated by serial photographic documentation of wound progression and longitudinal follow‐up throughout hospitalization.

Several limitations should be acknowledged. First, the relatively small sample size was partly attributable to recruitment disruptions during the COVID‐19 pandemic and the study design, which excluded outpatients. Second, convenience sampling at referral hospitals likely introduced selection bias, resulting in an overrepresentation of moderate‐to‐severe envenomation cases; conversely, patients with mild envenomation managed at primary healthcare facilities, or those who did not seek medical attention, may be underrepresented.

Finally, because laboratory investigations were performed according to clinical indication and resource availability, the assessment of systemic complications was incomplete. As specialized investigations for hemolysis, rhabdomyolysis, and organ injury were not routinely available, these findings should be interpreted with appropriate caution. As a result, these findings may not be fully generalizable to the broader snakebite population.

## 5. Conclusion

Snakebite envenomation caused by the zebra spitting cobra (*N. nigricincta nigricincta*) and the puff adder (*B. arietans*) remains an important cause of morbidity in Namibia, particularly among children and rural populations. Puff adder bites primarily resulted in localized tissue damage, but zebra spitting cobra envenomation exhibited more extensive necrosis, a greater need for surgical intervention, and more pronounced laboratory abnormalities suggestive of systemic involvement. No deaths occurred; however, prolonged hospitalization, surgical intervention, and amputation were all common outcomes.

This study provides baseline clinical evidence that may inform the development and refinement of national snakebite management guidelines in Namibia. Beyond clinical guidelines, reducing the burden of snakebite‐related morbidity will also require improved public awareness campaigns, timely referral pathways, better access to appropriate surgical care, and further research into region‐specific antivenom therapies.

## Author Contributions

Conceptualization: C.J.H. and K.N.; methodology: K.N. and C.J.H.; software: K.N. and C.J.H.; formal analysis: K.N. and E.N.; investigation: K.N. and C.J.H.; resources: K.N.; data curation: K.N.; writing—original draft preparation: K.N.; writing—review and editing: E.N., C.J.H., and K.N.; visualization: K.N.; supervision: C.J.H. and E.N.; project administration: K.N.

## Funding

No funding was received for this manuscript.

## Disclosure

All authors have read and agreed to the published version of the manuscript. An earlier version of this manuscript was included in the author′s academic thesis, available in the University of Namibia institutional repository [32].

## Conflicts of Interest

The authors declare no conflicts of interest.

## Data Availability

The data that support the findings of this study are available on request from the corresponding author. The data are not publicly available due to privacy or ethical restrictions.
